# A Self-Assembled Zn^II^-Nd^III^ Heterohexanuclear Dimer Based on a Hexadentate N_2_O_4_-Type Ligand and Terephthalic Acid: Synthesis, Structure, and Fluorescence Properties

**DOI:** 10.3390/molecules23071609

**Published:** 2018-07-02

**Authors:** Li-Jun Ru, Lei Gao, Wen-Ting Guo, Jian-Chun Ma, Wen-Kui Dong

**Affiliations:** 1 Chemical Engineering Department, Jiuquan Vocational Technical College, Jiuquan 735000, China; jqrlj@163.com (L.-J.R.); gwtzy044@126.com (W.-T.G.); 2 School of Chemical and Biological Engineering, Lanzhou Jiaotong University, Lanzhou 730070, China; GaoLei19910731@163.com (L.G.); majc0204@126.com (J.-C.M.)

**Keywords:** N_2_O_4_-type bisoxime, 3d-4f coordination compound, synthesis, crystal structure, fluorescence property

## Abstract

A self-assembled Zn^II^-Nd^III^ heterohexanuclear coordination compound [Zn_4_Nd_2_(L)_4_(bdc)_2_]·2NO_3_ based on a hexadentate Salamo-like chelating ligand (H_2_L = 1,2-bis(3-methoxysalicylideneaminooxy)ethane]) and H_2_bdc (H_2_bdc = terephthalic acid) has been synthesized and characterized by elemental analyses, IR and UV/Vis spectra, and X-ray crystallography. Two crystallographically equivalent [Zn_2_Nd(L)_2_] moieties lie in the inversion center linked by two (bdc)^2−^ ligands leading to a heterohexanuclear dimer in which the carboxylato group bridges the Zn^II^ and Nd^III^ atoms. The heteropolynuclear 3d-4f coordination compound includes four Zn^II^ atoms, two Nd^III^ atoms, four completely deprotonated (L)^2−^ units, two fully deprotonated (bdc)^2−^ units, and two crystalling nitrate ions. All of the Zn^II^ atoms in the Zn^II^-Nd^III^ coordination compound possess trigonal bipyramidal geometries and the Nd^III^ atoms possess distorted bicapped square antiprism coordination arrangements. In addition, the fluorescence properties of the ligand and the Zn^II^-Nd^III^ coordination compound were investigated.

## 1. Introduction

Salen-like and Salamo-like ligands have been extensively investigated in modern coordination chemistry for several decades [1,2], and their transition to metal coordination compounds have been widely investigated in electrochemical fields [3], biological fields [4], magnetic [5] and luminescent materials [6], and supramolecular buildings [7]. It is important to introduce suitable functional groups into the organic moiety of the ligands in order to improve or tune the properties of these metal coordination compounds [8].

Although these Salamo-like coordination compounds have been in the process of development, the heterometallic 3d-4f coordination compounds formed by terephthalic acid auxiliary ligands are still very rare. The multidentate chelating ligands may bring new changes to the structure and simple synthesis methods will play a catalytic role in the development of these coordination compounds [9,10]. The auxiliary ligands also play an important role in the construction of coordination compounds [11]. Some superior research on heterometallic coordination compounds with various functional properties have been reported [12,13,14,15].

In order to supplement and perfect the syntheses and structures of heterometallic 3d-4f coordination compounds in this study, we have synthesized a self-assembled Zn^II^-Nd^III^ hetero-metallic coordination compound [Zn_4_Nd_2_(L)_4_(bdc)_2_] by one-pot reaction of the ligand H_2_L with Zn^II^ acetate, Nd^III^ nitrate, and H_2_bdc in a 1:1:1:1 molar ratio. Furthermore, the fluorescence properties of the ligand H_2_L and its Zn^II^-Nd^III^ coordination compound were also studied.

## 2. Experimental

### 2.1. Materials and Physical Measurements

2-Hydroxy-3-methoxybenzaldehyde (>99%) was purchased from Alfa Aesar and used without further purification. The other reagents and solvents were analytical grade reagents from the Tianjin Chemical Reagent Factory.

C, H, and N analyses were obtained using a VarioEL V3.00 automatic elemental analysis instrument. Elemental analyses for Zn^II^ and Nd^III^ were detected by an IRIS ER/ S-WP-1 ICP atomic emission spectrometer. Melting points were obtained by using a microscopic melting point apparatus from the Beijing Taike Instrument Limited Company and the thermometer was uncorrected. IR spectra were recorded on a Vertex70 FT-IR spectrophotometer with samples prepared as KBr (400–4000 cm^–1^) pellets. UV/Vis absorption spectra were recorded on a Shimadzu UV-3900 spectrometer. Luminescence spectra in the solution were recorded on a Hitachi F-7000 spectrometer.

### 2.2. Synthesis of the Ligand H_2_L

**Scheme 1:**
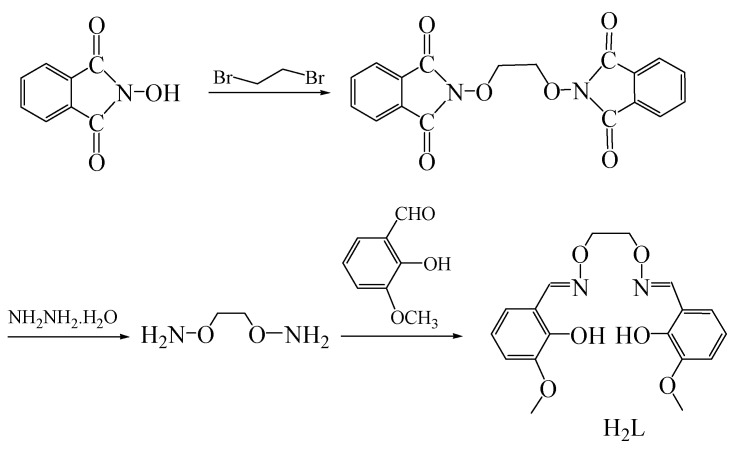
The synthetic route to H_2_L.

The main reaction steps involved in the synthesis of H_2_L are given in Scheme 1. H_2_L was synthesized according to the references reported earlier [16]. Yield: 74.2%. m.p. 131–133 °C. Anal. Calcd, for C_18_H_20_N_2_O_6_: C 59.99, H 5.59, N 7.77. Found: C 60.11, H 5.63, N 7.59.

### 2.3. Synthesis of the Zn^II^-Nd^III^ Coordination Compound

**Scheme 2:**
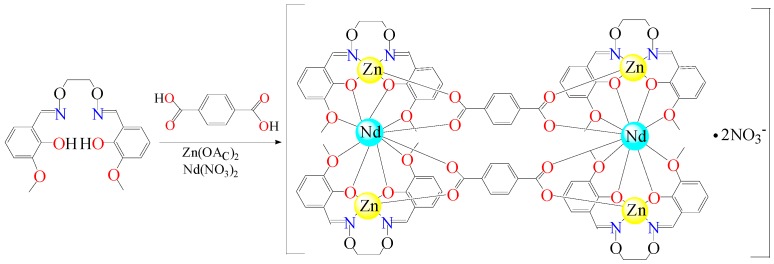
Assemble route for the Zn^II^-Nd^III^ coordination compound.

A mixture of H_2_L (36.0 mg, 0.1 mmol), Zn(OAc)_2_·2H_2_O (21.9 mg, 0.1 mmol), and Nd(NO_3_)_3_·6H_2_O (45.6 mg, 0.1 mmol) in chloroform and methanol (1:2 *v*/*v*, 3 mL) was stirred for 5 min at room temperature. Afterward, a solution of H_2_bdc (16.6 mg, 0.1 mmol) in DMF (1 mL) was added and the mixture was stirred for another 5 min at room temperature. The mixture solution was filtered and the filtrate was kept undisturbed in the dark to avoid decomposition of the Zn^II^-Nd^III^ building blocks. The yellow block-like crystals suitable for X-ray diffraction studies were obtained by vapor diffusion of diethyl ether into the filtrate for three days at room temperature. These were separated over filtration and air-dried before undergoing X-ray diffraction analysis (Scheme 2). The yield was 64.2%. Anal. Calcd. for C_88_H_80_N_10_Nd_2_Zn_4_O_38_ ([Zn_4_Nd_2_(L)_4_(bdc)_2_]·2NO_3_): C, 43.39; H, 3.31; N, 5.75; Nd, 11.84; Zn, 10.74. Found: C, 43.47; H, 3.49; N, 5.66; Nd, 11.71; Zn, 10.59.

### 2.4. X-ray Structure Determination of the Zn^II^-Nd^III^ Coordination Compound

The diffraction data was collected using graphite-monochromatized Mo K*α* radition (*λ* = 0.71073 Å) at 173(2) K. The structure was solved by using the program SHELXS-2016 and different Fourier techniques. It was refined by using the full-matrix least-squares method on *F*^2^ utilizing SHELXL-2016 [17,18]. The Zn^II^-Nd^III^ coordination compound contained large void and the solvent and the positive or negative ions located in the void could not be identified because it was highly disordered and had a small residual peak. Therefore, the SQUEEZE in PLATON program was performed to remove the highly disordered solvent and ions (Solvent Accessible Volume = 916, Electrons Found in S.A.V. = 136) [19]. All hydrogen atoms were added theoretically. The crystal and experimental data are shown in Table 1.

Crystallographic data have been deposited with the Cambridge Crystallographic Data Center as supplementary publication, No. CCDC 1843972 for the Zn^II^-Nd^III^ coordination compound. Copies of the data can be obtained free of charge on application to CCDC, 12 Union Road, Cambridge CB21EZ, UK (Telephone: +(44)-01223-762910; Fax: +44-1223-3360-33; E-mail: deposit@ccdc.cam.ac.uk). These data can be also obtained free of charge at www.ccdc.cam. ac.uk/conts/retrieving.htm.

## 3. Results and Discussion

### 3.1. Description of Crystal Structure of the Zn^II^-Nd^III^ Coordination Compound

**Figure 1:**
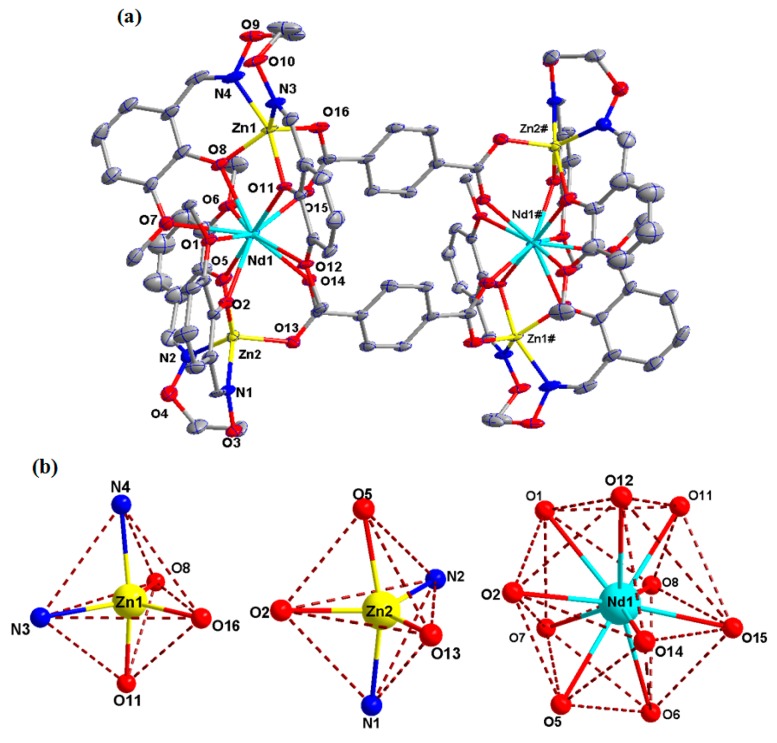
(**a**) Molecular structure and atom numberings of the Zn^II^-Nd^III^ coordination compound (hydrogen atoms are omitted for clarity); (**b**) Coordination polyhedra of the Zn^II^ and Nd^III^ atoms.

X-ray crystallographic analysis reveals the crystal structure of the Zn^II^-Nd^III^ coordination compound. Selected bond lengths and angles are given in Table S1. The molecular structure of the Zn^II^-Nd^III^ coordination compound is shown in Figure 1. The Zn^II^-Nd^III^ coordination compound crystallizes in the triclinic space group *P-*1 and consists of four Zn^II^ atoms, two Nd^III^ atoms, four completely deprotonated (L)^2−^ units, and two fully deprotonated (bdc)^2−^ units. Two crystallographically equivalent [Zn_2_Nd(L)_2_] moieties lie near the inversion center and are linked by two bdc^2−^ ligands. Each carboxylato group acts as a bridge between the Zn^II^ and Nd^III^ atoms. Each Zn^II^ atom has a penta-coordinated environment and adopts distorted trigonal bipyramidal geometries. The dihedral angles between the coordination planes and the benzene rings of terephthalic acid are 76.79(3)° and 82.49(3)°, respectively, which is shown in Figure S1. The carboxylato group bridges the Zn^II^ and Nd^III^ atoms in a *μ*_2_ fashion and each Nd^III^ atom is deca-coordinated and surrounded by ten oxygen atoms including four from the outer O_8_ cavity of the deprotonated ligand (L)^2−^ and two from the bdc^2−^ ligands. Therefore, the deca-coordinated Nd^III^ atom adopts a distorted bicapped square antiprism coordination arrangement. Moreover, this novel 4:4:2 ((L)^2−^:Zn(II):Ln(III)) heterometallic Salamo-type coordination compound possesses a symmetrical structure [12,13,14,15]. This is rarely reported in comparison to the 3d-4f Salamo-type coordination compound, which has the structures of 1:1:1 [20], 2:2:1 [9], and 2:2:2 [21].

### 3.2. Supramolecular Interaction of the Zn^II^-Nd^III^ Coordination Compound

As shown in Figure S2 and Table S2, there are abundant hydrogen bonds in the Zn^II^-Nd^III^ coordination compound including six formed pairs of intramolecular C1-H1B···O8, C9-H9B···O13, C18-H18B···O15, C19-H19B···O2, C27-H27B···O16 and C36-H36B···O14 hydrogen bonds. In addition, as shown in Figure S3, the space skeleton of the Zn^II^-Nd^III^ coordination compound adopts a 1D supramolecular structure by the action of C8-H8···O17, C8-H8···O19 and C39-H39···O17 hydrogen bonding [22].

### 3.3. IR Spectra of H_2_L and Its Zn^II^-Nd^III^ Coordination Compound

The FT-IR spectra of H_2_L and its corresponding Zn^II^-Nd^III^ coordination compound exhibits various bands in the 4000–400 cm^−1^ region (Figure S4). A typical C=N stretching band of the free ligand H_2_L appears at 1601 cm^−1^ and the Zn^II^-Nd^III^ coordination compound is observed at 1607 cm^−1^. The changes in the spectrum of the Zn^II^-Nd^III^ coordination compound show that H_2_L has coordinated with Zn(II) atoms. In addition, the free ligand H_2_L exhibits a typical Ar–O stretching frequency at 1258 cm^−1^ while the Ar–O stretching frequency of the Zn^II^-Nd^III^ coordination compound appears at 1240 cm^−1^. The shifts of Ar-O stretching frequencies show the Zn-O or Nd-O bond formation between Zn^II^ or Nd^III^ atoms and oxygen atoms of phenolic groups [23].

### 3.4. UV/Vis Absorption Spectra of H_2_L and Its Zn^II^-Nd^III^ Coordination Compound

The UV/Vis absorption spectra of the ligand H_2_L and its Zn^II^-Nd^III^ coordination compound in the dichloromethane solutions (1.0 × 10^−5^ mol·L^−1^) at 298 K are shown in Figure S5. Clearly, the absorption spectrum of the free ligand H_2_L consists of three relatively intense absorption peaks centered at ca. 223 nm, 269 nm, and 317 nm. The absorption peaks at 223 nm and 269 nm can be assigned to the π–π* transition of the benzene rings and the absorption peak at 317 nm can be attributed to the intra-ligand n–π* transition of the C=N bonds [23]. Upon coordination of the ligand H_2_L, the π–π* transitions of the phenyl rings in the Zn^II^-Nd^III^ coordination compound is bathochromically shifted to 233 nm and 280 nm, which indicates the coordination of Zn(II) and Nd(III) atoms with the ligand units, respectively [23]. Compared with the free ligand H_2_L, the absorption peak at 317 nm shifted to 375 nm from the UV/Vis spectrum of the Zn^II^-Nd^III^ coordination compound, which indicates that the oxime nitrogen atoms are involved in coordination with the Zn(II) atoms.

### 3.5. Fluorescence Properties of H_2_L and Its Zn^II^-Nd^III^ Coordination Compound

**Figure 2:**
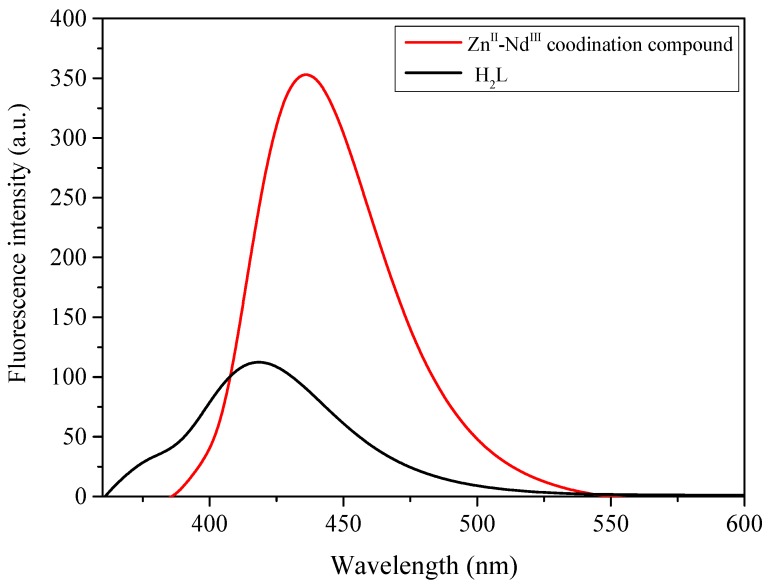
Fluorescence spectra of H_2_L and its corresponding Zn^II^-Nd^III^ coordination compound.

The fluorescent properties of H_2_L and its Zn^II^-Nd^III^ coordination compound were measured at room temperature (Figure 2). The Zn^II^-Nd^III^ coordination compound displays enhanced emission intensities compared to the corresponding ligand (H_2_L) when excited at 392 nm. The enhancement of fluorescence is due to the coordination of metal ions with the ligands. The ligand H_2_L exhibits a broad emission at 418 nm upon excitation at 392 nm, which should be assigned to the intraligand π–π* transition [24]. The Zn^II^-Nd^III^ coordination compound shows a higher intense photoluminescence with a maximum emission at 435 nm upon excitation at 392 nm. The emission peak positions of the Zn^II^-Nd^III^ coordination compound are similar to those of the free ligand H_2_L. The emission peaks in the spectrum of the Zn^II^-Nd^III^ coordination compound may also rise from the intra-ligand transition. Compared with the emission spectrum of H_2_L, enhanced fluorescence intensity of the Zn^II^-Nd^III^ coordination compound is observed, which indicates that the intra-ligand transition has been influenced due to the introduction of Zn(II) ions in the structure [24]. No emissions originating from metal-centered or metal-to-ligand/ligand-to-metal charge-transfer excited states are expected for the Zn^II^-Nd^III^. The emission observed in the Zn^II^-Nd^III^ coordination compound is tentatively assigned to the (π–π*) intra-ligand fluorescence.

## 4. Conclusions

In conclusion, we discovered a new Zn^II^-Nd^III^ heterometallic coordination compound [Zn_4_Nd_2_(L)_4_(bdc)_2_]·2NO_3_ with a Salamo-like chelating ligand H_2_L. The Zn^II^-Nd^III^ coordination compound contains four Zn^II^ atoms, two Nd^III^ atoms, four completely deprotonated (L)^2−^ units, and two fully deprotonated (bdc)^2−^ units. All of the Zn^II^ atoms in the Zn^II^-Nd^III^ coordination compound possesses trigonal bipyramidal geometries and the Nd^III^ atoms possesses a distorted bicapped square antiprism coordination arrangement. Furthermore, the fluorescent properties ofH_2_L and its Zn^II^-Nd^III^ coordination compound have also been discussed.

## Figures and Tables

**Table 1:** Crystal data and structure refinement for the Zn^II^-Nd^III^ coordination compound.

Coordination Compound	Zn^II^-Nd^III^
Empirical Formula	C_88_H_80_N_10_Nd_2_Zn_4_O_38_
Molecular Weight	2435.58
Color	colorless
Crystal Size, mm	0.270 × 0.250 × 0.220
Habit	Block-shaped
Crystal System	Triclinic
Space Group	*P*-1
Unit Cell Dimension	
*a*, Å	12.5088(9)
*b*, Å	16.0858(12)
*c*, Å	16.2996(12)
*α*, °	69.0298(19)
*β*, °	83.1411(18)
*γ*, °	87.649(2)
Volume, Å^3^	3040.6(4)
*Z*	1
Calculated Density, mg·m^–3^	1.330
Absorption Coefficient, mm^–1^	1.688
*F*(000)	1222
*θ* Range for Data Collection, °	1.346 to 27.756
*h*/*k*/*l* (min, max)	−16, 16/−14, 21/−20, 21
Reflections Collected	25,768
Completeness	98.5%
Data*/*Restraints*/*Parameters	13,926/3/644
Final *R* Indices [*I >* 2*σ*(*I*)]	*R*_1_ = 0.0422, *wR*_2_ = 0.1254
*R* Indices (All Data)	*R*_1_ = 0.0606, *wR*_2_ = 0.1294
Largest Diff. Peak and Hole (e·Å^–3^)	1.033, −0.898

## Supplementary Materials

Figure S1: View of the dihedral angles between the benzene rings of terephthalic acid and the basal planes (N_2_O_2_ planes) of the Zn^II^-Nd^III^ coordination compound. Figure S2: Intermolecular hydrogen bonding interactions of the Zn^II^-Nd^III^ coordination compound (hydrogen atoms, except those forming hydrogen bonds, are omitted for clarity). Figure S3: View of the 1D supramolecular structure of the Zn^II^-Nd^III^ coordination compound showing the C-H···O hydrogen bondings. Figure S4: IR spectra of H_2_L and its corresponding Zn^II^-Nd^III^ coordination compound. Figure S5: UV/Vis absorption spectra of H_2_L and its Zn^II^-Nd^III^ coordination compound. Table S1: Selected bond lengths (Å) and angles (°) for the Zn^II^-Nd^III^ coordination compound. Table S2: Hydrogen bonding interactions [Å, deg] for the Zn^II^-Nd^III^ coordination compound.
