# Supplementary material for: A Self-Assembled ZnII-NdIII Heterohexanuclear Dimer Based on a Hexadentate N2O4-Type Ligand and Terephthalic Acid: Synthesis, Structure, and Fluorescence Properties
Source: Molecules. 2018 Jul 2;23(7):1609. doi: 10.3390/molecules23071609 (PMC6099668; doi:10.3390/molecules23071609)
Supplement: Supplementary file 1 [file molecules-23-01609-s001.pdf]

A Self-assembled Zn^{II}-Nd^{III} Heterohexanuclear Dimer Based on a Hexadentate N_2O_4-type Ligand and Terephthalic Acid: Synthesis, Structure, and Fluorescence Properties

Li-Jun Ru[1], Lei Gao[2], Wen-Ting Guo[1], Jian-Chun Ma[2], Wen-Kui Dong[*,2]

[1] *Chemical Engineering Department, Jiuquan Vocational Technical College, Jiuquan 735000, China;*

[2] *School of Chemical and Biological Engineering, Lanzhou Jiaotong University, Lanzhou, Gansu 730070, PR China*

Supporting Information

Table S1 Selected bond lengths (Å) and angles (°) for the Zn^{II}-Nd^{III} coordination compound.

Table S2 Hydrogen bonding interactions [Å, deg] for the Zn^{II}-Nd^{III} coordination compound.

Fig. S1. View of the dihedral angles between the benzene rings of terephthalic acid and the basal planes (N_2O_2 planes) of the Zn^{II}-Nd^{III} coordination compound.

Fig. S2. Intermolecular hydrogen bonding interactions of the Zn^{II}-Nd^{III} coordination compound (hydrogen atoms, except those forming hydrogen bonds, are omitted for clarity).

Fig. S3. View of the 1D supramolecular structure of the Zn^{II}-Nd^{III} coordination compound showing the C-H···O hydrogen bondings.

Fig. S4. IR spectra of H_2L and its corresponding Zn^{II}-Nd^{III} coordination compound.

Fig. S5. UV/Vis absorption spectra of H_2L and its Zn^{II}-Nd^{III} coordination compound.

*Prof. Dr. Wen-Kui Dong, E-Mail: dongwk@126.com.

Table S1 Selected bond lengths (Å) and angles (°) for the Zn^{II}-Nd^{III} coordination compound.

Bonds Lengths (Å)		Bonds Lengths (Å)		Bonds Lengths (Å)	
Zn(1)-O(8)	2.014(3)	Zn(1)-O(11)	2.062(3)	Zn(1)-O(16)	1.988(3)
Zn(1)-N(3)	2.043(4)	Zn(1)-N(4)	2.139(4)	Zn(2)-N(1)	2.119(4)
Zn(2)-N(2)	2.043(4)	Zn(2)-O(2)	2.004(3)	Zn(2)-O(5)	2.067(3)
Zn(2)-O(13)	1.979(3)	Nd(1)-O(15)	2.422(3)	Nd(1)-O(14)	2.443(3)
Nd(1)-O(2)	2.454(3)	Nd(1)-O(11)	2.466(3)	Nd(1)-O(8)	2.454(3)
Nd(1)-O(5)	2.479(3)	Nd(1)-O(1)	2.647(3)	Nd(1)-O(7)	2.683(3)
Nd(1)-O(6)	2.781(3)	Nd(1)-O(12)	2.802(3)		

Angles (°)		Angles (°)		Angles (°)	
O(16)-Zn(1)-O(8)	112.59(12)	O(16)-Zn(1)-N(3)	120.06(14)	O(8)-Zn(1)-N(3)	126.84(13)
O(16)-Zn(1)-O(11)	97.99(11)	O(8)-Zn(1)-O(11)	126.84(13)	N(3)-Zn(1)-O(11)	86.75(13)
O(16)-Zn(1)-N(4)	97.95(17)	O(8)-Zn(1)-N(4)	86.75(13)	N(3)-Zn(1)-N(4)	92.70(15)
O(11)-Zn(1)-N(4)	161.97(14)	O(13)-Zn(2)-O(2)	111.87(12)	O(13)-Zn(2)-N(2)	117.47(15)
O(2)-Zn(2)-N(2)	130.04(14)	O(13)-Zn(2)-O(5)	97.38(12)	O(2)-Zn(2)-O(5)	79.50(11)
N(2)-Zn(2)-O(5)	86.84(15)	O(13)-Zn(2)-N(1)	98.33(14)	O(2)-Zn(2)-N(1)	87.43(13)
N(2)-Zn(2)-N(1)	92.79(16)	O(5)-Zn(2)-N(1)	162.46(13)	O(15)-Nd(1)-O(14)	73.41(9)
O(15)-Nd(1)-O(2)	150.62(9)	O(14)-Nd(1)-O(2)	77.31(9)	O(15)-Nd(1)-O(11)	70.90(9)
O(14)-Nd(1)-O(11)	107.98(9)	O(2)-Nd(1)-O(11)	117.05(9)	O(15)-Nd(1)-O(8)	77.39(9)
O(14)-Nd(1)-O(8)	150.70(10)	O(2)-Nd(1)-O(8)	131.95(9)	O(11)-Nd(1)-O(8)	63.62(10)
O(15)-Nd(1)-O(5)	107.68(10)	O(14)-Nd(1)-O(5)	70.88(10)	O(2)-Nd(1)-O(5)	63.87(10)
O(11)-Nd(1)-O(5)	178.47(9)	O(8)-Nd(1)-O(5)	116.84(10)	O(15)-Nd(1)-O(1)	139.04(9)
O(14)-Nd(1)-O(1)	125.60(9)	O(2)-Nd(1)-O(1)	60.44(8)	O(11)-Nd(1)-O(1)	68.64(9)
O(8)-Nd(1)-O(1)	79.32(9)	O(5)-Nd(1)-O(1)	112.83(9)	O(15)-Nd(1)-O(7)	125.36(10)
O(14)-Nd(1)-O(7)	140.48(9)	O(2)-Nd(1)-O(7)	80.01(9)	O(11)-Nd(1)-O(7)	111.09(9)
O(8)-Nd(1)-O(7)	59.18(9)	O(5)-Nd(1)-O(7)	70.15(9)	O(1)-Nd(1)-O(7)	66.12(9)
O(15)-Nd(1)-O(6)	66.54(11)	O(14)-Nd(1)-O(6)	96.94(10)	O(2)-Nd(1)-O(6)	120.23(10)
O(11)-Nd(1)-O(6)	121.04(9)	O(8)-Nd(1)-O(6)	68.70(9)	O(5)-Nd(1)-O(6)	58.40(10)
O(1)-Nd(1)-O(6)	132.67(9)	O(7)-Nd(1)-O(6)	67.69(10)	O(15)-Nd(1)-O(12)	97.33(10)
O(14)-Nd(1)-O(12)	67.57(9)	O(2)-Nd(1)-O(12)	68.80(9)	O(11)-Nd(1)-O(12)	58.03(8)
O(8)-Nd(1)-O(12)	119.41(9)	O(5)-Nd(1)-O(12)	121.95(9)	O(1)-Nd(1)-O(12)	66.18(9)
O(7)-Nd(1)-O(12)	131.35(9)	O(6)-Nd(1)-O(12)	160.92(9)		

Table S2 Hydrogen bonding interactions [Å, deg] for the Zn^{II}-Nd^{III} coordination compound.

D-H ···A	d(D-H)	d(H ···A)	d(D ···A)	∠DHA
C1-H1B ···O8	0.98	2.58	3.328(5)	133
C9-H9B ···O13	0.99	2.39	3.315(6)	155
C18-H18B ···O15	0.98	2.51	3.095(7)	118
C19-H19B ···O2	0.98	2.55	3.319(6)	135
C27-H27B ···O16	0.99	2.41	3.313(5)	152
C36-H36B ···O14	0.98	2.50	3.127(6)	122
C8-H8 ···O17	0.95	2.07	2.822(10)	135
C8-H8 ···O19	0.95	2.50	3.402(9)	159
C39-H39 ···O17	0.95	2.58	3.317(10)	135

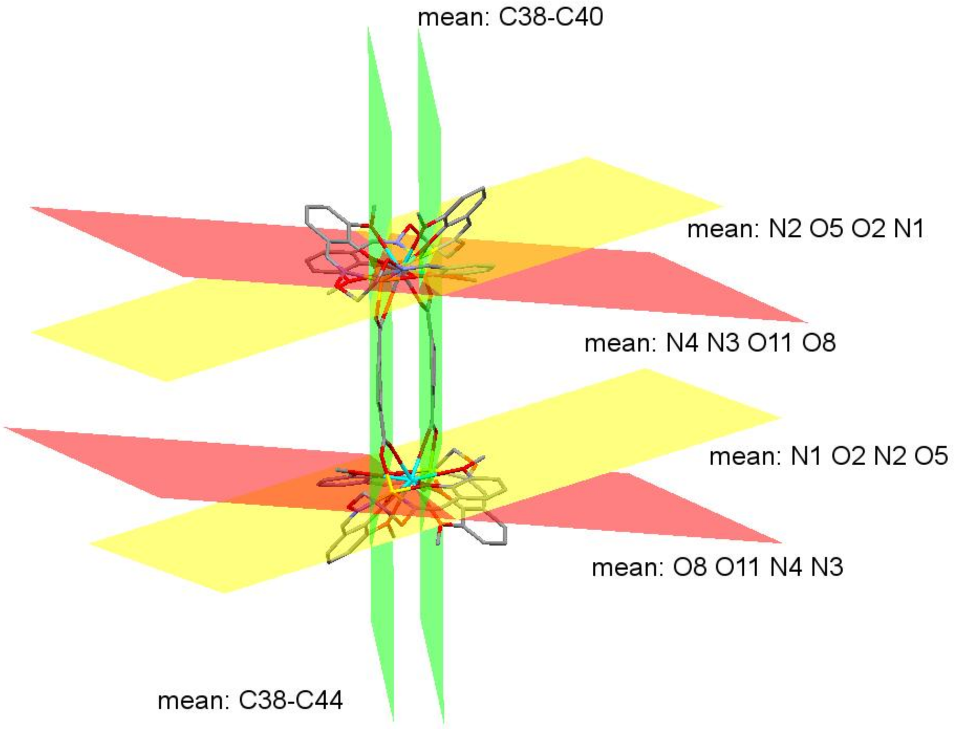

Fig. S1. View of the dihedral angles between the benzene rings of terephthalic acid and the basal planes (N_2O_2 planes) of the Zn^{II}-Nd^{III} coordination compound.

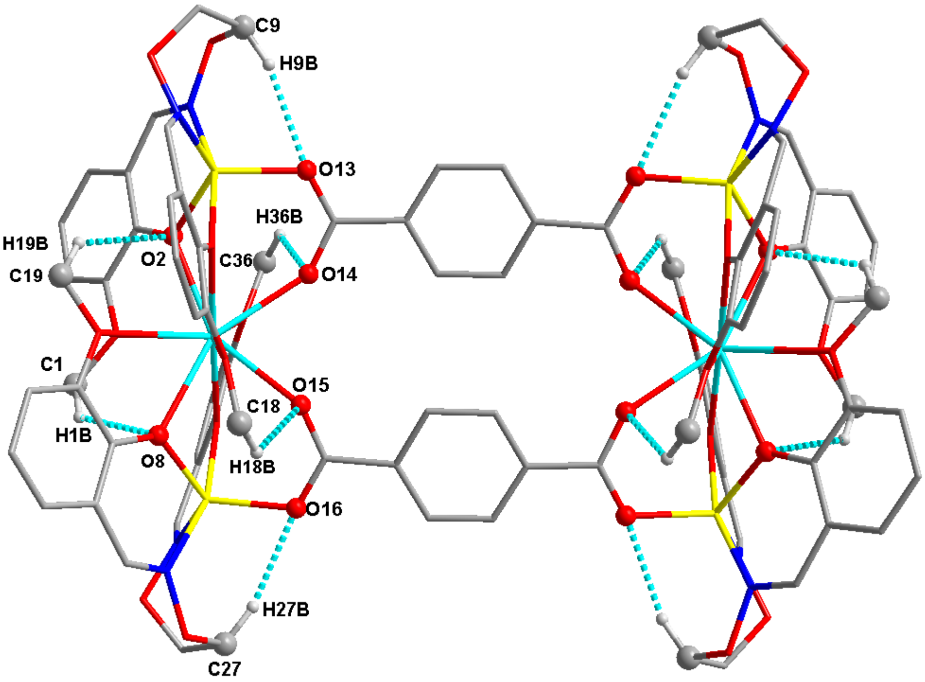

Fig. S2. Intermolecular hydrogen bonding interactions of the Zn^{II}-Nd^{III} coordination compound (hydrogen atoms, except those forming hydrogen bonds, are omitted for clarity).

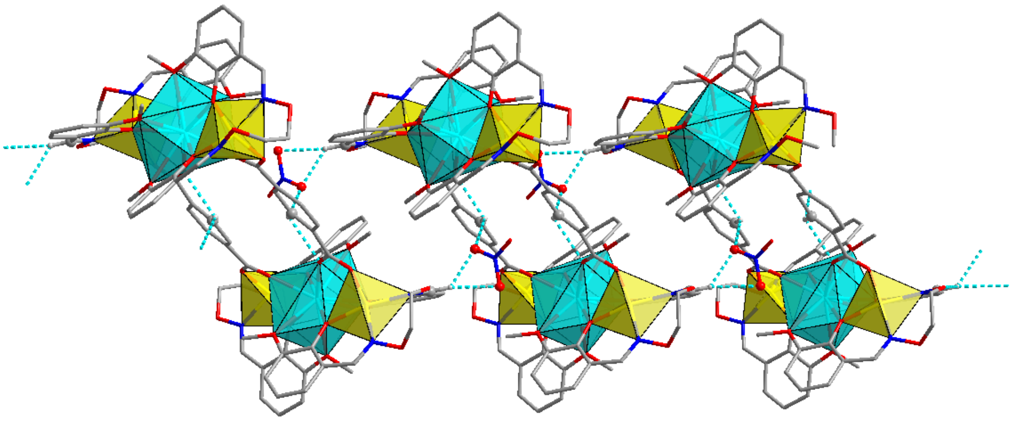

Fig. S3. View of the 1D supramolecular structure of the Zn^{II}-Nd^{III} coordination compound showing the C-H ···O hydrogen bondings.

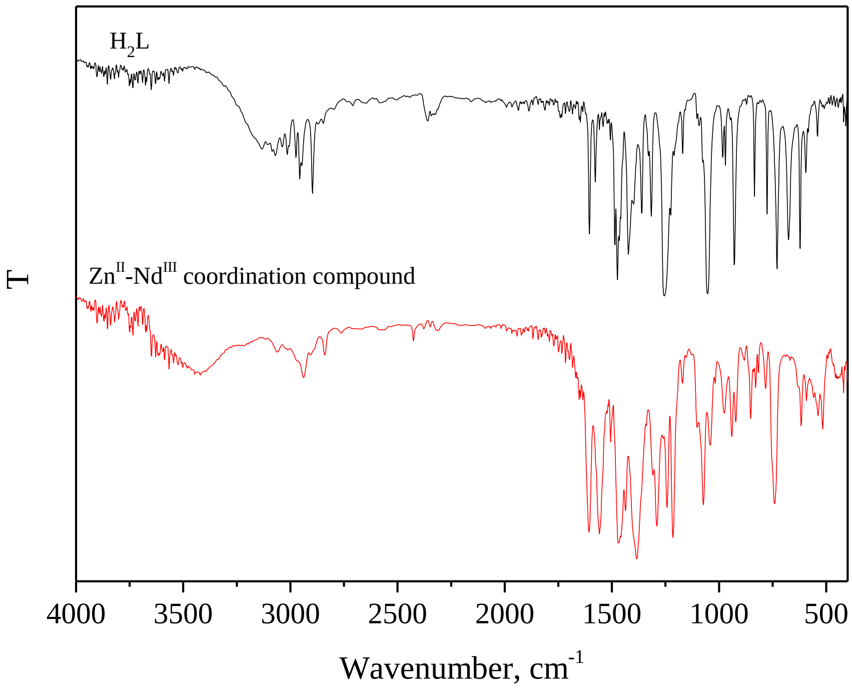

Fig. S4. IR spectra of H_2L and its corresponding Zn^{II}-Nd^{III} coordination compound.

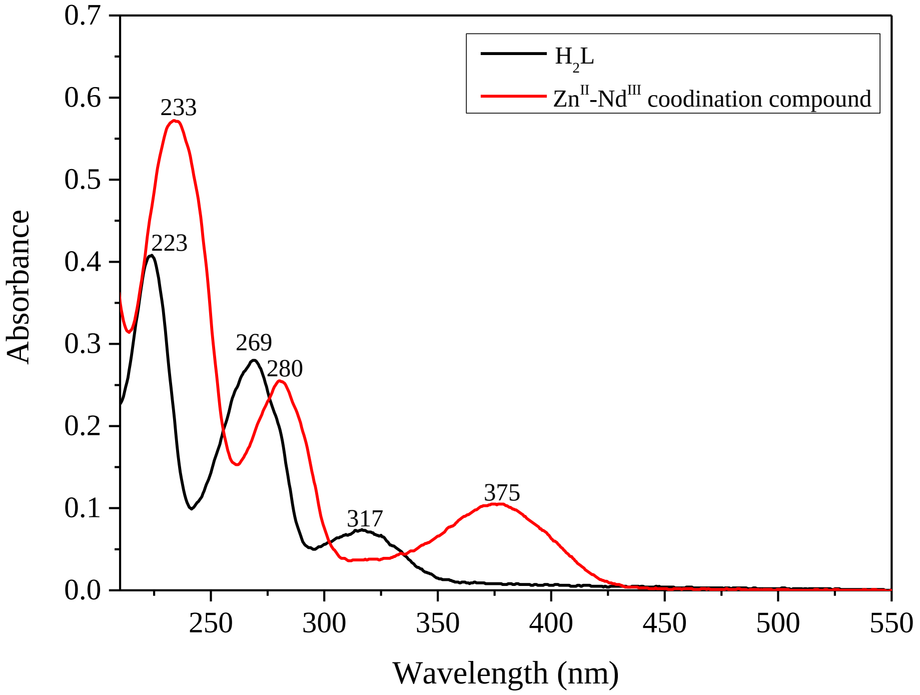

Fig. S5. UV/Vis absorption spectra of H_2L and its Zn^{II}-Nd^{III} coordination compound.
